# Remission of CVB3-induced myocarditis with Astragaloside IV treatment requires A20 (TNFAIP3) up-regulation

**DOI:** 10.1111/jcmm.12459

**Published:** 2015-03-01

**Authors:** Jun Gui, Ruizhen Chen, Wei Xu, Sidong Xiong

**Affiliations:** a Institute for Immunobiology, Shanghai Medical College, Fudan UniversityShanghai, China; b Key Laboratory of Viral Heart Diseases, Shanghai Institute of Cardiovascular Diseases, Zhongshan Hospital, Fudan UniversityShanghai, China; c Institutes of Biology and Medical Sciences, Soochow UniversitySuzhou, China

**Keywords:** Astragaloside IV, Viral myocarditis, CVB3, NF-κB, A20

## Abstract

Viral myocarditis (VMC) most prevalently caused by coxsackievirus B3 (CVB3) infection is characterized by severe cardiac inflammation. Therapeutic options for the disease are still limited. Astragaloside IV (AST-IV), a purified small molecular saponin (C_41_H_68_O_14_, MW 784), is the main active component of Chinese medical herb Astragalus which has been empirically prescribed for the treatment of heart dysfunction for centuries. In this study, we investigated the effect of AST-IV on CVB3-induced myocarditis and explored its possible mechanism involved. The results showed that AST-IV administration alleviated the severity of myocarditis and attenuated cardiac inflammation, which was mediated by inhibition of nuclear factor-kappaB (NF-κB) signalling. Importantly, we further identified that the inhibitory effect of AST-IV on NF-κB signalling was through increasing A20 (TNFAIP3) expression. Moreover, we validated that A20 was critical for the therapeutic efficacy of AST-IV on CVB3-induced myocarditis. Finally, we revealed that AST-IV enhanced A20 expression at post-transcriptional level by stabilization of mRNA. Our findings uncover a previously unknown mechanism for AST-IV in the treatment of VMC because of modulating inflammatory response *via* increasing A20 expression, which provide a potential target for screening new drugs and are helpful for optimization of the therapeutic strategies for VMC.

## Introduction

Viral myocarditis (VMC) characterized by myocardial inflammation represents one of the most challenging clinical problems in cardiology, associated with a broad spectrum of pathological triggers and a wide range of clinical presentations that vary from mild dyspnoea to acute heart failure. It has been estimated to account for up to 12% of sudden death in patients under 40 years of age and become a leading cause of dilated cardiomyopathy which may result in death for 50% of patients 1–2 years after diagnosis 1–3. The dominant aetiology of VMC is considered to be the enteroviruses of picornavirus family, with coxsackievirus B3 (CVB3) being the most common one, and the same virus strain induced similar inflammatory heart disease in susceptible strains of mice 4,5. The exact mechanisms of pathological damage of heart induced by VMC are not well understood. The likely mechanisms involve immune-mediated and direct viral cytotoxicity, especially the reactive inflammatory responses which significantly contribute to cardiac damage and ensuing morbidity 6.

To date, there is no specific therapy for VMC patients, though extensive investigations on therapeutic approaches have been conducted. The conventional treatment of symptoms in VMC is by using beta-blockers, calcium channel blockers, diuretics or angiotensin converting enzyme inhibitors 7. Several proposed clinical treatment strategies that target specific points were reported, including the application of immunosuppressive agents (azathioprine, prednisone and cyclosporine) 8–10, intravenous immunoglobulin which may replace antibodies, neutralize pathogens and enhance clearance of inflammatory cytokines that contribute to myocytes destruction 11,12, and antiviral agents, such as interferons and pleconaril, which may target the causative organism, possibly halting the cascade of myocyte destruction 13–15.

In addition to that, Traditional Chinese Medicine (TCM) which has been used for many centuries in China plays an increasingly important role in clinical treatment. Astragalus, the root extracts from the Chinese herbal plant *Astragalus membranaceus*, is a staple of TCM. It was primarily prescribed for the treatment of ‘*Xin Ji*’ which referred to ‘palpitation’ in TCM 16,17. Modern pharmacological studies have demonstrated that Astragalus shows effective in protecting myocardium and improving cardiac function in the treatment of heart failure 18–20. The natural Astragalus contains multiple ingredients, some of which may cause side effects for patients. Thus, it's much necessary to study the effects and underlying mechanisms of active constituents of Astragalus.

**Fig 1:**
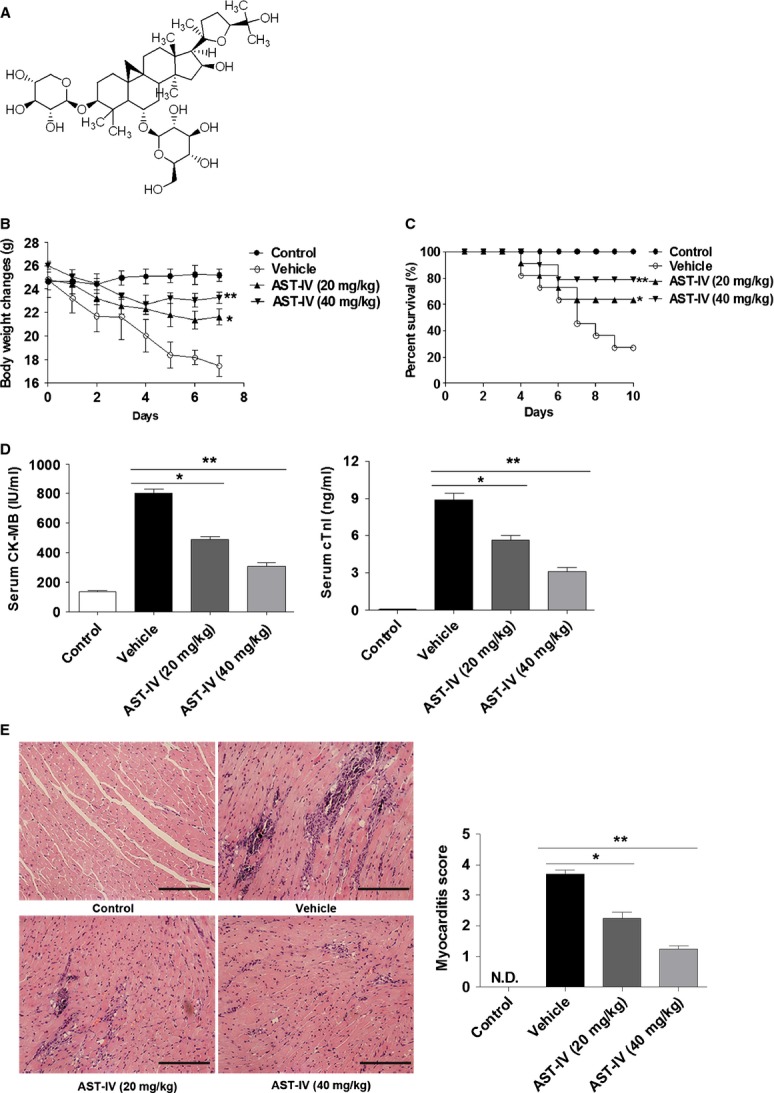
AST-IV administration mediated protection against CVB3-induced myocarditis. (A) The chemical structure of AST-IV. (B–E) Male BALB/c mice were administered intraperitoneal injection with 10^3^ TCID_50_ CVB3 at day 0. Mice without inoculation were used as normal controls. The infected mice were received two doses AST-IV (20 and 40 mg/kg/day) intragastric injections immediately after CVB3 inoculation. The bodyweight change (*n* = 8; B) and survival rate (*n* = 15 per group; C) were, respectively, monitored daily until day 7 and day 10 post infection. Serological indices of myocarditis, CK-MB activity and cTnI level in serum were detected on day 7 post infection (*n* = 8; D). Paraffin sections of heart tissues were prepared on day 7 and cardiac inflammation was revealed by haematoxylin and eosin staining (magnification: ×200, scale bars: 100 μm). The severity of myocarditis was scored by a standard 0–4 scale according to the foci of mononuclear infiltration and myocardial necrosis (*n* = 8; E). Individual experiments were conducted three times with similar results, with one representative shown for each group. Data show the means ± SEM. *, *P* < 0.05; **, *P* < 0.01; N.D., not detected.

Astragaloside IV (AST-IV), 3-0-beta-Dxylopyranosyl-6-0-beta-d-glucopyranos- ylcycloastra-genol (Fig.1A), one of the major active components of Astragalus, is a purified small molecular saponin (C_41_H_68_O_14_, MW 784) 21–23. Experimental studies showed that AST-IV could improve cardiac function, inhibit compensatory hypertrophy of myocardial cells and lower the number of apoptotic myocytes 24–26. The positive effects of AST-IV on attenuating chronic myocardial fibrosis and treating VMC have been somewhat characterized. Previous work showed that AST-IV could attenuate myocardial fibrosis by inhibiting TGF-β1 signalling in CVB3-induced cardiomyopathy and exert antiviral effects against CVB3 by up-regulating expression of IFN-γ mRNA 27,28. However, whether AST-IV can find clinical application in treating VMC still needs more investigation, and the underlying mechanism remains largely unknown, though regarding the immunological mechanism of AST-IV has been carried out from cellular and molecular levels with some progress that immune modulation was reported to be involved in 29,30.

In this study, we examined the effect of AST-IV on CVB3-induced myocarditis and explored its potential molecular mechanism involved.

## Materials and methods

### Mice

Pathogen-free Male BALB/c mice (H-2^d^), 6 weeks of age were purchased from Shanghai Experimental Animal Center of Chinese Academy of Sciences. Animal experiments were performed according to the National Institutes of Health Guide for Care and Use of Laboratory Animals. The experimental procedures were approved by the Shanghai Medical Laboratory Animal Care and Use Committee (Permit number: SYXK 2011-0038) as well as the Ethical Committee of Fudan University (Permit number: 2011016). All surgery carried out in this study was strictly performed in a manner to minimize suffering of laboratory mice.

### Virus

CVB3 (Nancy strain) was maintained by passage through HeLa cells (ATCC number: CCL-2). Viral titre was routinely determined prior to infection by a 50% tissue culture infectious dose (TCID_50_) assay of HeLa cell monolayer.

### Cell culture

HeLa cells were cultured in DMEM (Invitrogen Life Technologies, Gaithersburg, MD, USA) supplemented with 10% Fetal Bovine Serum (FBS) (Invitrogen Life Technologies) in a 5% CO_2_ incubator at 37°C. Cardiac myocytes were isolated from neonatal mice within 72 hrs of birth as previously reported 31.

### Astragaloside IV treatment

Astragaloside IV was purchased from Shanghai Hisanns Biotechnology Company, Shanghai, China (purity up to 99.5%). For *in vivo* use, it was dissolved in twice-distilled water, with the help of 5% carboxymethyl cellulose-sodium. Mice were inoculated intraperitoneally with 10^3^ TCID_50_ CVB3 to establish VMC model. For the drug treatment groups, mice were intragastrically injected with AST-IV daily at the dose of 20 or 40 mg/kg immediately after virus infection. Infected mice without AST-IV treatment were daily administrated with the same volume of vehicle. Mice without CVB3 inoculation were used as normal controls. Serum and heart tissue were collected at the indicated time-points (*n* = 6–8 per group). Survival rates of mice in each group were monitored till day 10 post infection (*n* = 15 per group).

For *in vitro* experiments, AST-IV was dissolved in DMSO. Cardiac myocytes (1 × 10^5^ per well) cultured in 12-well plates were pre-treated with 10 μM AST-IV for 24 hrs, then exposed to CVB3 for the indicated time. Cells cultured without viral infection and AST-IV treatment served as normal controls. Infected cells without AST-IV treatment were used as infected controls.

### Tissue histopathology and myocarditis grading

Seven days following CVB3 infection, the heart tissues were collected, sectioned and stained with haematoxylin and eosin. The myocarditis score was assessed by previously described 0–4 scale 32, in which 0 = no inflammation; 1 = one to five distinct mononuclear inflammatory foci with involvement of 5% or less of the cross-sectional area; 2 = more than five distinct mononuclear inflammatory foci, or involvement of over 5% but not over 20% of the cross-sectional area; 3 = diffuse mononuclear inflammation involving over 20% of the area, without necrosis; and 4 = diffuse inflammation with necrosis.

### Immunohistochemistry

Cardiac sections at day 7 were immunostained with anti-CD3 (eBioscience, San Diego, CA, USA) and anti-CD11b (BD, Franklin Lakes, NJ, USA). Five complete sections were analysed per mouse. Quantification was determined by counting five different fields per section.

### Real-time polymerase chain reaction

Total RNA was extracted by Trizol reagent (Invitrogen) according to the manufacturer's instructions. The first-strand cDNA was synthesized using oligo(dT) primers and the RevertAid™ M-MuLV transcription enzyme (Fermentas, Pittsburgh, PA, USA). Samples were subjected to real-time RT-PCR analysis on a Lightcycler480 (Roche Diagnostics, Mannheim, Germany) with SYBR Green system (Takara, Biotechnology, Dalian, China) using the following parameters: 2 min. at 50°C followed by 10 min. at 95°C and 40 cycles of 15 sec. at 95°C and 1 min. at 60°C. Gene expression levels were normalized to that of housekeeping gene *GAPDH*. Primers were as follows: *A20* Sense: 5′-CTAAGCCAACGAGTAGGTTCTGTG-3′, Antisense: 5′-CCATACA TCTGCTTGAACTGGTAG-3′; *GAPDH* Sense: 5′-CTCTGGAAAGCTGTGG CGTGATG-3′, Antisense: 5′-ATGCCAGTGAGCTTCCCGTTCAG-3′.

### Adenovirus/lentivirus production and intravenous administration

The adenovirus and lentivirus were produced as previously described 33. Briefly, adenoviruses encoding A20 (Ad-A20) and the control (Ad-LacZ) were created using the Virapower adenovirus expression system according to the manufacturer's instructions (Invitrogen). To overexpress A20 *in vivo*, mice received intravenous injection of 50 μl of adenovirus Ad-A20 or the control Ad-LacZ (3 × 10^9^ plaque forming units, pfu) 2 days before CVB3 infection. The lentiviruses encoding shRNA for knocking down A20 (LV-shA20) and the control (LV-ctrl) were generated by co-transfection of three plasmids pLKO.1-shA20/pLKO.1-scramble shRNA, psPAX2 and pMD2.G (Addgene, Cambridge, MA, USA) into 293T cells using Lipofectamine Plus (Invitrogen). Viral supernatants were collected and concentrated by ultracentrifugation (Beckman, Coulter Commercial Enterprise, Shanghai, China). To knock down A20 *in vivo*, mice received intravenous injection of 100 μl of lentivirus LV-shA20 or the control LV-ctrl (2 × 10^7^ pfu) 2 days before CVB3 infection. Mice were intragastrically administrated with 20 mg/kg/day AST-IV in adenovirus injected mice and administrated with 40 mg/kg/day AST-IV in lentivirus injected mice immediately after CVB3 inoculation. Serum and heart tissue were collected at the indicated time-points (*n* = 6–8 per group). Survival rates of mice in each group were monitored till day 10 post infection (*n* = 20 per group).

### Cytokine assays

Levels of TNF-α, IL-1β, IL-6 and MCP-1 in serum and heart homogenates were determined by ELISA (eBioscience) following the manufacturers' instructions.

### Western blot

Forty micrograms (μg) of extracted protein were fractionated by 8–10% SDS-PAGE. The blot was probed with 1 μg/ml primary antibody for p-ERK/ERK, p-JNK/JNK, p-p38/p38, p-IKKβ/IKKβ, p-p65/p65, p-IκBα/IκBα (Cell Signaling Technology, Boston, MA, USA), β-actin, A20 (Santa Cruz Biotechnology, Dallas, Texas, USA). HRP-conjugated anti-rabbit or antimouse IgG (Southern Biotech, Birmingham, Alabama, USA) was used as a secondary antibody.

### Nuclear factor-kappaB DNA binding activity

Nuclear protein was isolated from cardiac tissues using the Nuclear and Cytoplasmic Extraction Reagents Kit (Thermo Scientific™ NE-PER™, Pittsburgh, PA, USA). Nuclear factor-kappaB (NF-κB) DNA binding activity in nuclei was determined using the NF-κB p65 transcription factor assay kit according to the manufacturer's instructions (Cayman Chemical, Ann Arbor, Michigan, USA). Briefly, 10 μg nuclear extracts were added to the designated wells with complete transcription factor binding assay buffer (100 μl/well) and incubated overnight at 4°C. After five times washing with 200 μl wash buffer, 100 μl of 1:100 diluted NF-κB p65 antibody was added for 1 hr at room temperature. After washing, 100 μl of 1:100 diluted goat anti-rabbit HRP conjugate was added to each well. 45 min. later, 100 μl of transcription factor developing solution was added to each well and incubated for 15 min. without light. Finally, 100 μl of stop solution was added and absorbance was measured at 450 nm.

### Echocardiography

Mice were anaesthetized (2% isoflurane) and echocardiographic examination was performed by transthoracic echocardiography with a Vevo 770 scanner (Visual Sonics, Toronto, Ontario, Canada). LV diameters at end-diastole (LVEDd), end-systole (LVEDs), septal wall thickness (SWd), posterior wall thickness in end diastole (PWd), fractional shortening (FS) and ejection fraction value (EF) was calculated.

### Statistical analysis

All data were expressed as means ± SEM or from a representative experiment of three independent experiments. Comparisons between different treatment groups were performed by one-way anona, followed by the *post hoc* analysis using Fisher's least significant difference method for intergroup comparisons. The statistical significance was presented as *P < 0.05, **P < 0.01, and ***P < 0.001.

## Results

### AST-IV treatment alleviated CVB3-induced myocarditis

We investigated the effect of AST-IV on CVB3-induced myocarditis. The results showed that CVB3 infected mice without AST-IV treatment underwent a dramatic and continuous loss of bodyweight as maximal to 29.7%, and more than 70% mice died within 10 days post infection. On the contrary, mice receiving AST-IV administration had a little fluctuation in bodyweight and a significantly improved survival rate (20 mg/kg 63.64%, 40 mg/kg 78.75%, Fig.1B and C). Consistently, serological indices of CK-MB activities and cTnI levels were significantly decreased in mice with AST-IV treatment compared with vehicle treated mice (20 mg/kg; *P* < 0.05, 40 mg/kg; *P* < 0.01, Fig.1D), indicating a significantly reduced myocardial injury. Histological analysis of heart sections revealed that vehicle group mice with CVB3 infection developed severe myocarditis on day 7 with diffuse inflammation, whereas AST-IV treatment led to a significant remission of myocarditis showing few restricted mononuclear inflammation foci and tiny necrosis (20 mg/kg; *P* < 0.05, 40 mg/kg; *P* < 0.01, Fig.1E). The high dose drug group (40 mg/kg) showed more efficient therapeutic effect on CVB3-induced myocarditis.

Cardiac function was measured by echocardiography 7 days after CVB3 infection. The cardiac functional parameters were shown in the Table1. There was no significant difference in heart rate (HR, beat per minute) between different groups. Posterior wall in diastole (PWd), LV end-diastolic diameter (LVEDd) and LV end-systolic dimension (LVEDs) was much lower, and LV ejection fraction (LVEF) and FS was much higher in AST-IV treated mice when compared with vehicle group mice, indicating that the cardiac function was significantly improved after AST-IV treatment.

**Table 1:** Cardiac function of CVB3 mice with AST-IV treatment

	Control (*n* = 8)	Vehicle (*n* = 6)	AST-IV (20 mg/kg) (*n* = 6)	AST-IV (40 mg/kg) (*n* = 8)
PWd (mm)	0.54 ± 0.023	0.73 ± 0.049	0.68 ± 0.024†,*	0.63 ± 0.032‡,**
SWd (mm)	0.943 ± 0.14	1.052 ± 0.057	1.012 ± 0.205	0.976 ± 0.0042
LVEDd (mm)	3.52 ± 0.14	4.24 ± 0.13	3.862 ± 0.173†,*	3.660 ± 0.058‡,**
LVEDs (mm)	2.032 ± 0.25	2.76 ± 0.376	2.28 ± 0.26†,**	2.148 ± 0.02‡,**
EF (%)	65.06 ± 1.20	45.24 ± 2.70	53.65 ± 2.12†,*	56.43 ± 1.33‡,**
FS (%)	36.57 ± 1.75	21.89 ± 1.43	25.84 ± 2.15†,*	28.92 ± 1.26‡,**
HR (bpm)	443 ± 32	407 ± 35	398 ± 30	404 ± 27

All values are means ± SEM.

† Vehicle Group *versus* AST-IV (20 mg/kg) Group.

‡ Vehicle Group *versus* AST-IV (40 mg/kg) Group.

* *P* < 0.05

** *P* < 0.01.

All the above data indicated that AST-IV treatment could effectively protect mice from lethal myocarditis caused by CVB3 infection.

### AST-IV treatment attenuated pro-inflammatory response in CVB3 infected mice

**Fig 2:**
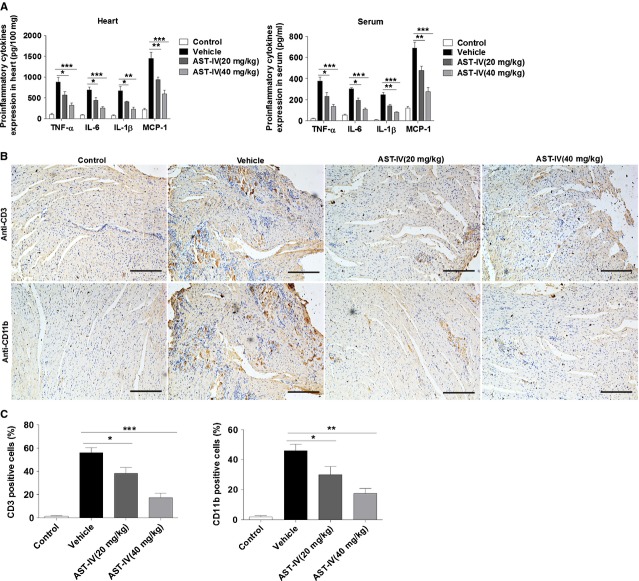
AST-IV treatment suppressed pro-inflammatory response in CVB3 infected mice. (A) Heart tissue homogenates and serum were harvested at day 7 following CVB3 infection. Protein levels of pro-inflammatory cytokines including TNF-α, IL-6, IL-1β and MCP-1 were determined by ELISA (*n* = 6). (B) Immunohistochemical analysis with cardiac sections of cardiac CD3 and CD11b expression at day 7 (scale bars: 100 μm). Individual experiments were conducted three times with similar results, with one representative shown for each group. (C) Quantitative analysis of CD3 positive cells and CD11b positive cells in cardiac sections (*n* = 6). Data show the means ± SEM. *, *P* < 0.05; **, *P* < 0.01; ***, *P* < 0.001.

Viral myocarditis is a serious clinical condition characterized by cardiac inflammation that contributes to the impaired cardiac function 34. We examined the effect of AST-IV treatment on the inflammatory response in CVB3 infected mice. The results showed that AST-IV treated mice had significantly lower expression levels of pro-inflammatory cytokines, including TNF-α, IL-1β, IL-6 and MCP-1 both in cardiac tissues and serum when compared with vehicle group mice (Fig.2A). Furthermore, the inflammatory cell infiltrates were characterized by immunohistochemical analyses. Cardiac sections at day 7 were immunostained with immune cells marker anti-CD3 and anti-CD11b. The results showed that upon infection the number of infiltrative CD3^+^ cells and CD11b^+^ cells were strongly increased within the myocardium. However, reduced recruitment of the inflammatory cells (Fig.2B and C) was observed in cardiac tissue of CVB3 mice with AST-IV treatment. Obviously, higher dose AST-IV treatment (40 mg/kg) showed more significantly decreasing recruitment of inflammatory cells. These results suggested that AST-IV treatment attenuated the activated inflammatory response in CVB3 infected mice by reducing pro-inflammatory cytokines expression and cardiac inflammatory cells infiltration, consequently preventing the cardiac damage caused by inflammation.

### NF-κB signalling was inhibited in CVB3 infected mice with AST-IV treatment

**Fig 3:**
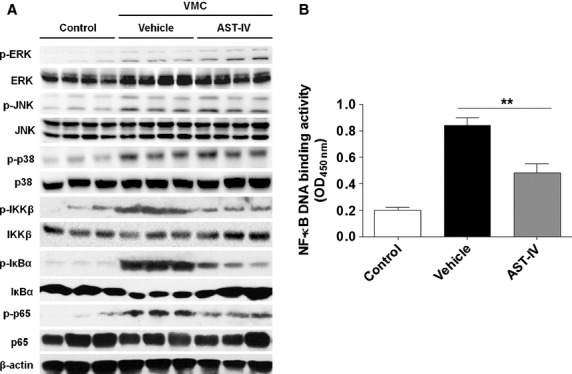
AST-IV treatment inhibited NF-κB signalling activation in CVB3 mice. (A) Male BALB/c mice were inoculated with 10^3^ TCID_50_ CVB3 at day 0, then intragastrically injected with 40 mg/kg/day AST-IV immediately after CVB3 inoculation. Mice without inoculation were used as normal controls. Heart homogenates were prepared on day 4. The phosphorylation of ERK, JNK, p38, IKKβ, p65 and IκBα were assessed by western blotting. The data were collected from three mice for each group. The assay was conducted three times with similar results, with one representative shown for each group. (B) NF-κB DNA binding activity was analysed by NF-κB p65 transcription factor assay kit (*n* = 6). Values were presented as the means ± SEM. **, *P* < 0.01.

The signalling pathways responsible for inflammatory response mainly include MAPK and NF-κB pathways 35. We investigated whether AST-IV would have effect on the activation of MAPK and NF-κB signalling. The results showed that there was a significant increase in the levels of the phosphorylation of ERK, JNK, P38, IKKβ, IκBα and p65-NF-κB subunit in CVB3 mice treated with vehicle. AST-IV treatment did not affect the phosphorylation of ERK, JNK and p38, but decreased the phosphorylation of IKKβ, IκBα and p65, indicating that AST-IV appeared to have no effect on MAPK signalling, but significantly inhibited NF-κB signalling activation in CVB3 mice (Fig.3A). We further measured the transcription factor binding activity of heart nuclear extracts to a NF-κB consensus sequence and found that NF-κB DNA binding activity was also significantly decreased after AST-IV treatment (*P* < 0.01, Fig.3B), which conferred lower expression of multiple pro-inflammatory genes that targeted by NF-κB. These data suggested that AST-IV effectively inhibited NF-κB signalling activation, thus contributing to the suppression of pro-inflammatory response in CVB3 infected mice.

### The inhibitory effect of AST-IV on NF-κB signalling was through increasing A20 expression

**Fig 4:**
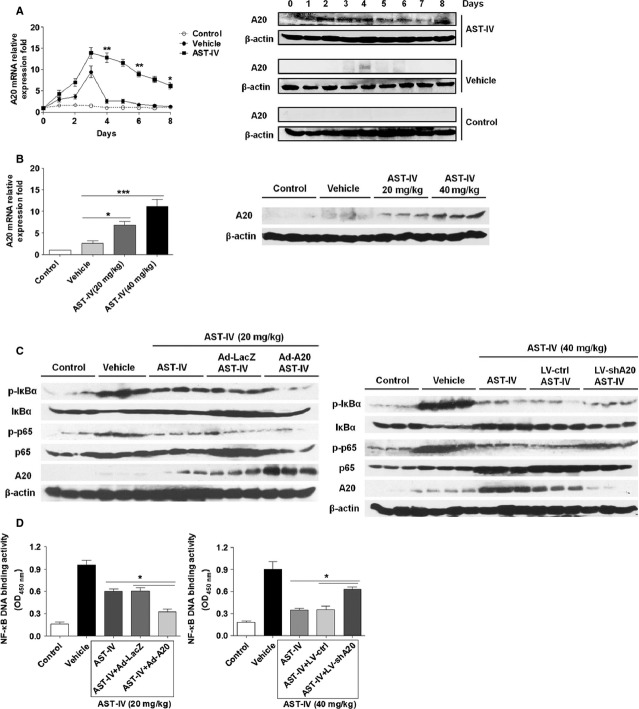
The inhibitory effect of AST-IV on NF-κB was through increasing A20 expression. (A) Cardiac A20 expression at different time-points in CVB3 mice with 40 mg/kg/day AST-IV treatment. Relative *A20*mRNA abundance was normalized to the expression of *GAPDH*. A20 protein expression was analysed by Western blot. β-actin was probed as the loading control. (B) Cardiac A20 expression at day 4 in CVB3 mice with two doses AST-IV (20 and 40 mg/kg/day) treatment. (C) Mice were intravenously injected with 3 × 10^9^ pfu of either Ad-A20 or Ad-LacZ to overexpress A20, or 2 × 10^7^ pfu of either LV-shA20 or LV-ctrl to knock down A20 2 days before 10^3^ TCID_50_ dose of CVB3 inoculation. The adenovirus injected mice were intragastrically administrated with 20 mg/kg/day AST-IV and the lentivirus injected mice were administrated with 40 mg/kg/day AST-IV. Heart homogenates were prepared on day 4 after CVB3 infection. The phosphorylation of p65 and IκBα were assessed by western blotting. The data were collected from three mice for each group. (D) NF-κB DNA binding activity was analysed by NF-κB p65 transcription factor assay kit (*n* = 6). All experiments were independently performed in triplicate. Values were presented as the means ± SEM. *, *P* < 0.05; **, *P* < 0.01; ***, *P* < 0.001.

The above data showed that AST-IV could inhibit IKK activation in NF-κB pathway, implying that AST-IV might function upstream of IKKα/β. Our previous work demonstrated that A20, also known as TNF-α induced protein 3 (TNFAIP3), was required to inhibit CVB3-induced IKK complex activation and phosphorylation of IκBα by restricting TNF receptor associated factor 6 (TRAF6) K63-linked ubiquitylation 33. Thus, we attempted to investigate whether A20 was involved in the inhibitory effect of AST-IV on NF-κB signalling. We analysed the mRNA and protein expression of A20 daily in the cardiac tissue of CVB3 mice with AST-IV treatment (40 mg/kg). Of interest, we found that AST-IV treatment resulted in high expression of A20 both in mRNA and protein level, as compared with normal mice and vehicle treated mice (Fig.4A). And the increased A20 expression in cardiac tissue with AST-IV treatment was in dose dependent manner (Fig.4B). These results indicated that AST-IV treatment could up-regulate cardiac A20 expression in CVB3 mice.

To investigate the effect of A20 on NF-κB signalling in CVB3 mice with AST-IV treatment, we overexpressed or knock down A20 expression *in vivo* by intravenously injected with adenovirus encoding A20 (Ad-A20/Ad-LacZ) and lentivirus knocking down A20 (LV-shA20/LV-ctrl) respectively 2 days before CVB3 inoculation. Mice were intragastrically administrated with low dose AST-IV (20 mg/kg/day) in adenovirus injected mice and administrated with high dose AST-IV (40 mg/kg/day) in lentivirus injected mice immediately after CVB3 inoculation. The cytoplasmic and nuclear protein was extracted from heart homogenates at day 4. The results showed that the phosphorylation levels of IκBα and p65-NF-κB subunit were more significantly lowered in A20 overexpression mice with low dose AST-IV treatment (20 mg/kg). By contrast, their phosphorylation levels were enhanced in A20 knock down mice with high dose AST-IV treatment (40 mg/kg; Fig.4C), indicating that A20 knock down could abrogate the inhibitory function of AST-IV on NF-κB signalling. The NF-κB DNA binding activity was also much more lower in A20 overexpressed mice and higher in A20 knock down mice with AST-IV treatment, in line with the above results (Fig.4D).

These data indicated that AST-IV inhibited NF-κB signalling in CVB3 mice, at least in part, through increasing A20 expression.

### A20 was required for anti-inflammation of AST-IV in CVB3 infected mice

**Fig 5:**
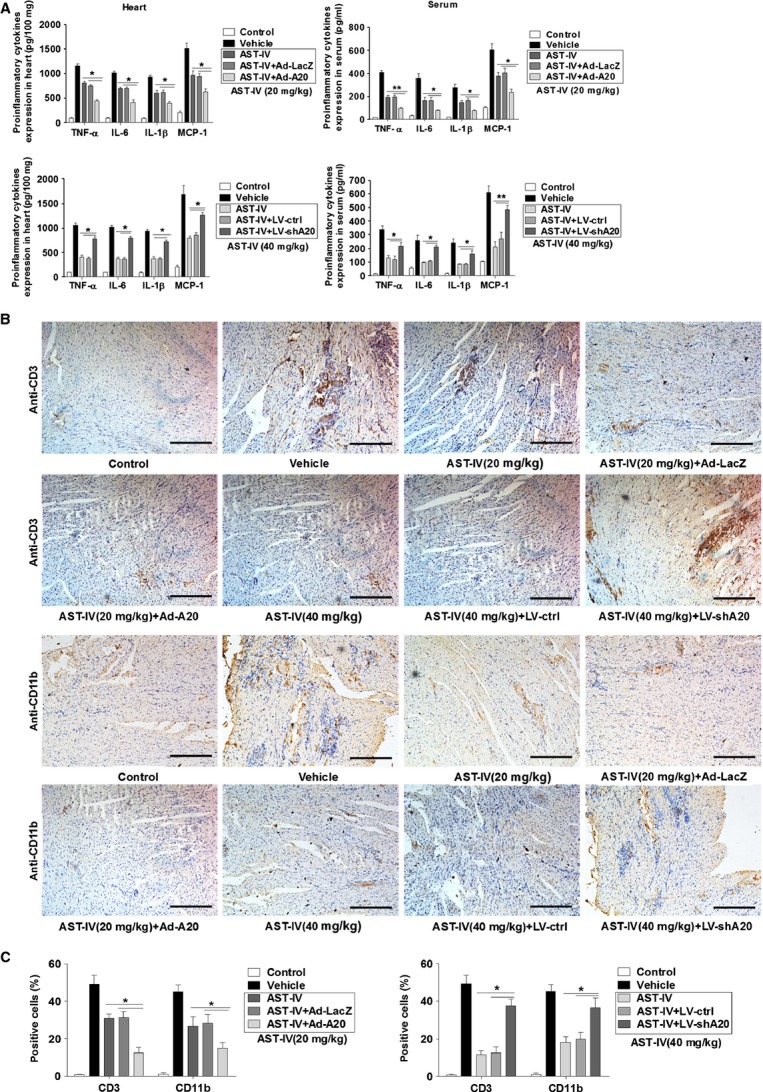
A20 expression was required for anti-inflammation of AST-IV. Mice were intravenously injected with 3 × 10^9^ pfu of either Ad-A20 or Ad-LacZ to overexpress A20, or 2 × 10^7^ pfu of either LV-shA20 or LV-ctrl to knock down A20 2 days before 10^3^ TCID_50_ dose of CVB3 inoculation. Mice without inoculation were used as normal controls. The CVB3 mice were intragastrically administrated with 20 mg/kg/day AST-IV in adenovirus injected mice and administrated with 40 mg/kg/day AST-IV in lentivirus injected mice immediately after CVB3 inoculation. (A) Heart homogenates and serum were harvested on day 7. Protein levels of pro-inflammatory cytokines including TNF-α, IL-6, IL-1β and MCP-1 were determined by ELISA (*n* = 6). (B) Immunohistochemical analysis with cardiac sections of CD3 and CD11b expression on day 7 (scale bars: 100 μm). Individual experiments were conducted three times with similar results, with one representative shown for each group. (C) Quantitative analysis of CD3 positive cells and CD11b positive cells in cardiac sections (*n* = 6). All the values were presented as the means ± SEM. *, *P* < 0.05; **, *P* < 0.01.

To examine the role of A20 in the pro-inflammatory response in CVB3 mice with AST-IV treatment, we overexpressed or knock down A20 *in vivo* as described above, then treated mice with AST-IV immediately after CVB3 inoculation. The results showed that much lower expression levels of the pro-inflammatory cytokines were observed in Ad-A20 injected mice when compared with Ad-LacZ injected mice with 20 mg/kg AST-IV treatment. By contrast, when A20 knock down, the cytokine expression levels were increased in CVB3 mice even with high dose AST-IV treatment (40 mg/kg; Fig.5A). The inflammatory cellular infiltrates were characterized by immunohistochemical analyses. In the presence of A20 overexpression, the CD3^+^ cells and CD11b^+^ cells were much more greatly decreased in CVB3 mice with 20 mg/kg AST-IV treatment. However, the attenuation of the recruitment of the inflammatory cells was abolished in A20 knock down mice even with high dose AST-IV treatment (40 mg/kg; Fig.5B and C). These results suggested that A20 was required for the anti-inflammation effect of AST-IV in CVB3 infected mice.

### A20 was critical for the therapeutic effect of AST-IV on CVB3-induced myocarditis

**Fig 6:**
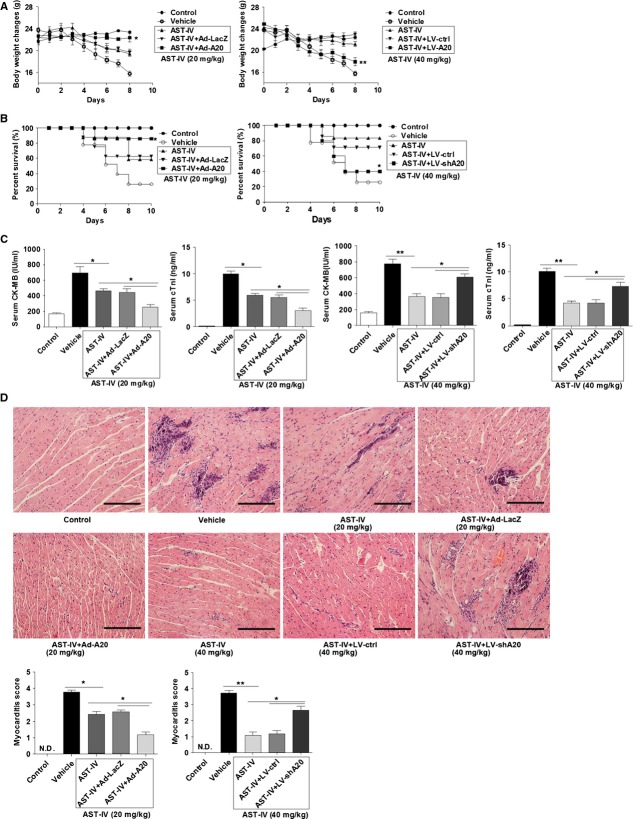
A20 was critical for the therapeutic effect of AST-IV on myocarditis. Mice were intravenously injected with 3 × 10^9^ pfu of either Ad-A20 or Ad-LacZ to overexpress A20, or 2 × 10^7^ pfu of either LV-shA20 or LV-ctrl to knock down A20 2 days before 10^3^ TCID_50_ dose of CVB3 inoculation. The CVB3 mice were intragastrically administrated with 20 mg/kg/day AST-IV in adenovirus injected mice and administrated with 40 mg/kg/day AST-IV in lentivirus injected mice immediately after CVB3 inoculation. (A and B) The bodyweight change (*n* = 7; A) and survival rate (*n* = 20 per group; B) were, respectively, monitored daily until day 8 and day 10 post infection. (C) Serological indices of myocarditis, the activity of CK-MB and cTnI in serum were detected on day 7 post-infection (*n* = 7). (D) Paraffin sections of heart tissues were prepared on day 7 and cardiac inflammation was revealed by haematoxylin and eosin staining (magnification: ×200, scale bars: 100 μm). The severity of myocarditis was scored by a standard 0–4 scale according to the foci of mononuclear infiltration and myocardial necrosis (*n* = 7). Individual experiments were conducted three times with similar results, with one representative shown for each group. Data show the means ± SEM. *, *P* < 0.05; **, *P* < 0.01; N.D., not detected.

We next investigated the role of A20 in the therapeutic effect of AST-IV on CVB3-induced myocarditis. The results showed that in the low dose (20 mg/kg) AST-IV treated CVB3 mice, A20 overexpressed mice could more efficiently prevent mice from bodyweight loss when compared with its control Ad-LacZ injected mice (*P* < 0.05). And a higher survival rate was observed in Ad-A20 injected mice when compared to Ad-LacZ mice with 20 mg/kg AST-IV treatment (85.7% *versus* 62.5%, *P* < 0.05). On the contrary, A20 knock down could attenuate the protective effect of AST-IV against bodyweight loss and LV-shA20 injected mice still led to a persistent and significant weight loss even treated with 40 mg/kg AST-IV. The survival rate of A20 knock down mice with 40 mg/kg AST-IV treatment was decreased to 40% and much lower than AST-IV treated mice (83.3%) or LV-ctrl injected mice (71.4%; *P* < 0.05; Fig.6A and B). Consistent with that, CK-MB activities and cTnI levels were more significantly decreased in Ad-A20 injected mice than Ad-LacZ mice with 20 mg/kg AST-IV treatment (*P* < 0.05), and more increased in LV-shA20 injected mice than LV-ctrl mice with 40 mg/kg AST-IV treatment (*P* < 0.05; Fig.6C), indicating the critical role of A20 in AST-IV preventing myocardial injury. Histological analysis of heart sections at day 7 revealed that A20 overexpression led to a more improved relief of myocardial inflammation showing few restricted mononuclear inflammation foci and no necrosis when compared to mice with 20 mg/kg AST-IV treatment or plus Ad-LacZ delivery (myocarditis score 1.07 ± 0.06 *versus* 2.17 ± 0.17, 2.33 ± 0.17, *P* < 0.05). However, more severe damage of heart tissue was founded in A20 knock down mice mediated by LV-shA20 compared with LV-ctrl group mice received 40 mg/kg AST-IV treatment, evidenced by more increasing inflammation and necrosis lesions (myocarditis score 3.25 ± 0.25 *versus* 1.35 ± 0.15, *P* < 0.05; Fig.6D), indicating that A20 knock down could abrogate the therapeutic effect of AST-IV.

We also measured the cardiac function of each group mice by echocardiography on day 7. Much lower LVEDd (mm; *P* < 0.05) and LVEDs (mm; *P* < 0.05), and higher EF (%; *P* < 0.01) and FS (%; *P* < 0.05) were observed in A20 overexpressed mice plus received 20 mg/kg AST-IV treatment, when compared to mice with drug treatment only or plus Ad-LacZ delivery (Table2), indicating that A20 could enhance the improvement of cardiac function in the condition of low dose AST-IV treatment. However, PWd (mm) and LVEDd (mm) was increased in A20 knock down mice received 40 mg/kg AST-IV treatment compared with the control group (LV-ctrl injected mice; 0.708 ± 0.033 *versus* 0.63 ± 0.05, 3.660 ± 0.083 *versus* 3.318 ± 0.19; *P* < 0.05). EF (%) and FS (%) were significantly decreased (53.80 ± 1.61 *versus* 60.43 ± 1.33, 24.63 ± 4.5 *versus* 29.09 ± 1.46; *P* < 0.05; Table3), indicating a worse cardiac function when A20 knock down even in high dose AST-IV treatment.

**Table 2:** Cardiac function of Ad-LacZ/Ad-A20 injected CVB3 mice with AST-IV treatment

	Control (*n* = 8)	Vehicle (*n* = 6)	AST-IV (20 mg/kg) (*n* = 6)	AST-IV + Ad-LacZ (20 mg/kg) (*n* = 7)	AST-IV + Ad-A20 (20 mg/kg) (*n* = 8)
PWd (mm)	0.52 ± 0.039	0.73 ± 0.059	0.62 ± 0.019†,*	0.63 ± 0.057	0.59 ± 0.034
SWd (mm)	0.928 ± 0.13	1.046 ± 0.097	0.988 ± 0.13	0.976 ± 0.097	0.964 ± 0.105
LVEDd (mm)	3.572 ± 0.13	4.104 ± 0.13	3.572 ± 0.13†,*	3.710 ± 0.18	3.458 ± 0.15‡,*
LVEDs (mm)	2.456 ± 0.23	2.76 ± 0.376	2.456 ± 0.23†,*	2.56 ± 0.326	2.354 ± 0.28‡,*
EF (%)	64.26 ± 1.31	46.24 ± 3.70	53.06 ± 1.31†,*	51.24 ± 3.70	60.65 ± 3.12‡,**
FS (%)	35.97 ± 1.45	20.90 ± 1.83	24.97 ± 1.45†,*	23.90 ± 1.83	30.84 ± 2.25‡,*
HR (bpm)	413 ± 25	367 ± 37	403 ± 25	397 ± 37	408 ± 32

All values are means ± SEM.

† AST-IV (20 mg/kg) Group *versus* Vehicle Group.

‡ AST-IV + Ad-LacZ Group *versus* AST-IV + Ad-A20 Group.

* *P* < 0.05

** *P* < 0.01.

**Table 3:** Cardiac function of LV-ctrl/LV-shA20 injected CVB3 mice with AST-IV treatment

	Control (*n* = 8)	Vehicle (*n* = 6)	AST-IV (40 mg/kg) (*n* = 6)	AST-IV + LV-ctrl (40 mg/kg) (*n* = 6)	AST-IV + LV-shA20 (40 mg/kg) (*n* = 8)
PWd (mm)	0.52 ± 0.039	0.73 ± 0.059	0.60 ± 0.035†,*	0.63 ± 0.050	0.708 ± 0.033‡,*
SWd (mm)	0.928 ± 0.13	1.046 ± 0.097	0.972 ± 0.105	0.996 ± 0.0021	1.110 ± 0.056
LVEDd (mm)	3.572 ± 0.13	4.104 ± 0.13	3.462 ± 0.122†,**	3.318 ± 0.19	3.660 ± 0.083‡,*
LVEDs (mm)	2.456 ± 0.23	2.76 ± 0.376	2.054 ± 0.28†,**	2.148 ± 0.09	2.296 ± 0.13
EF (%)	64.26 ± 1.31	46.24 ± 3.70	56.65 ± 3.12†,**	60.43 ± 1.33	53.80 ± 1.61‡,*
FS (%)	35.97 ± 1.45	20.90 ± 1.83	28.84 ± 2.25†,**	29.09 ± 1.46	24.63 ± 4.5‡,*
HR (bpm)	413 ± 25	367 ± 37	398 ± 32	404 ± 27	396 ± 24

All values are means ± SEM.

† AST-IV (40 mg/kg) Group *versus* Vehicle Group.

‡ AST-IV + LV-ctrl Group *versus* AST-IV + LV-shA20 Group.

* *P* < 0.05

** *P* < 0.01.

All these data suggested that A20 was critical for the therapeutic effect of AST-IV on CVB3-induced myocarditis.

### AST-IV increased A20 expression by stabilization of mRNA

**Fig 7:**
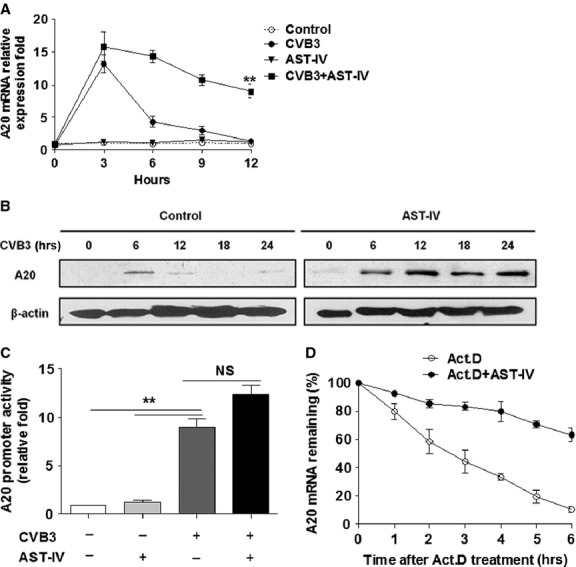
AST-IV increased A20 expression by stabilizing mRNA. (A) AST-IV (10 μM) pre-treated cardiac myocytes for 24 hrs, then exposed to CVB3 infection for the indicated time. At the time-points, cells were harvested with Trizol. The mRNA level of *A20* was analysed by real-time PCR normalized to *GAPDH*. (B) The protein level of A20 was assay by Western blot. β-actin was probed as the loading control. (C) Cardiac myocytes transfected with pA20-luc were treated with AST-IV or not, then infected with CVB3 for 3 hrs. Cells were lysed and subjected to luciferase assay. (D) Cardiac myocytes were infected with CVB3 for 3 hrs, exposed to actinomycin D (Act.D, 3 μg/ml) for 0–6 hrs in the absence or presence of AST-IV. Levels of *A20*mRNA were determined by real-time PCR. Each assay was independently performed in triplicate. Data were presented as the means ± SEM. **, *P* < 0.01.

We next addressed how AST-IV could up-regulate A20 expression. We examined the effect of AST-IV on A20 expression *in vitro*. Interestingly, we observed that A20 mRNA could be induced after CVB3 infection, but subsequently decreased quickly, implying that A20 was transiently induced following CVB3 infection. Of note, the infected cells with AST-IV treatment sustained the elevation of the steady-state A20 expression, but AST-IV per se could not induce A20 mRNA expression (Fig.7A). Accordingly, the A20 protein expression showed similar phenomenon (Fig.7B). Generally, steady-state gene expression is a coordination of transcription and decay of mRNA. To investigate whether AST-IV had effect on A20 transcriptional induction, we detected the A20 promoter activity by performing a reporter assay with pA20-Luc. The results showed that A20 promoter was remarkably activated after CVB3 infection. However, no significant change was viewed with AST-IV treatment (Fig.7C), indicating that AST-IV had no effect on A20 mRNA transcription. Indeed, existing evidence has demonstrated that A20 is an immediate-early NF-κB target gene 36. On the basis of that, we inferred that CVB3 infection activated NF-κB, which lead to the transcription of A20 immediately. But the mRNA of A20 was unstable and degraded quickly. AST-IV may stabilize A20 mRNA. To examine this possibility, cells were pre-treated with CVB3 for 3 hrs to induce A20, then treated with actinomycin D, an inhibitor of RNA synthesis, in the presence or absence of AST-IV for an additional 1–6 hrs. The results showed that A20 mRNA was rapidly reduced by the treatment of actinomycin D. In the presence of AST-IV, A20 mRNA was much more stable (Fig.7D). The above data suggested AST-IV sustained the stability of A20 mRNA, which leaded to increased mRNA accumulation and protein induction. Therefore, AST-IV promoted A20 expression post-transcriptionally by stabilizing mRNA.

## Discussion

Astragalus, the dried root of a low shrub *Astragalus membranaceus*, has long been used for the treatment of heart dysfunction in TCM. Currently, Astragalus remedies are the commonly used Chinese patent medicine to improve cardiac functions for the clinical treatment of various cardiovascular disorders in China. Over 25% of the 90 recipes in the 2010 edition of the Chinese Pharmacopoeia for cardiovascular diseases contain Astragalus 16,20. AST-IV is the main active component of Astragalus. Previous reports showed that AST-IV could attenuate myocardial fibrosis by inhibiting TGF-β1 signalling, mediate protection against cardiac hypertrophy *via* inhibiting the Ca^2+^/CaN signalling, and reduce cardiac ischaemia/reperfusion injury *via* energy regulation 27,37,38. In this study, we investigated the effect of AST-IV on VMC and explored the possible molecular mechanism. Mice were intraperitoneally infected with CVB3 to establish VMC model, immediately followed by daily AST-IV administration. The therapeutic effect was carefully investigated. The results showed that AST-IV administration could efficiently prevent CVB3 mice from bodyweight loss, increase mice survival rate, relief myocardial injury and improve cardiac function in dose-dependent manner, which suggested that AST-IV could exert its therapeutic effects since the early stage of VMC progression, though the evident symptoms have not yet showed up. Nevertheless, in the clinical setting, most of the patients receive treatment when they are suffering from heart failure symptoms. Thus, for the future study to further test the therapeutic effect of AST-IV on VMC, AST-IV may be administrated to mice 3 or 4 days after CVB3 infection at which the infected mice begin to look sick. Overall, our data indicated that AST-IV may represent a potential therapeutic agent for VMC and provided evidence for its possible extension to clinical trials.

Viral myocarditis is characterized by excessive inflammation of myocardium leading to heart injury. Cytokines and chemokines produced by cardiomyocytes and infiltrated inflammatory cells over the course of virus infection are important for the disease progression. Virus infection in cardiac myocytes initiated the expression of pro-inflammatory cytokines including TNF-α, IL-6, IL-1β and chemokines. These cytokines are crucial for the recruitment and activation of immune cells which accumulate in the infected heart and strongly augment the expression of pro-inflammatory cytokines, result in the massive inflammation and aggravated injury in heart 6,34. Here, we found that AST-IV treatment significantly reduced pro-inflammatory cytokines expression levels both in cardiac tissues and serum, and inflammatory cells infiltration. Our data suggested that AST-IV treatment modulated inflammatory response in VMC, consequently preventing the cardiac damage caused by inflammation. Interestingly, we observed that myocardial virus titre was reduced in AST-IV treated mice when compared with vehicle group mice (data not shown), which may also contribute to the therapeutic effect of AST-IV on VMC, consistent with the previous report which showed that AST-IV decreased virus titres of CVB3 in primarily cultured myocardial cells 28. Other studies elucidated the therapeutic mechanisms of AST-IV for the treatment of cardiovascular diseases, implicating in its ability to improve cardiac function by antioxidant and anti-apoptosis 26,39. Of course, we do not exclude these mechanisms which may also involve in the therapeutic effect of AST-IV on VMC.

Virus infection leads to signalling cascades, most commonly NF-κB and MAPK pathways, which are responsible for transcription of pro-inflammatory genes. Our results showed that AST-IV treatment significantly inhibited CVB3-induced NF-κB signalling activation rather than MAPK pathway. Other research groups reported that AST-IV could inhibit lipopolysaccharide or high glucose induced NF-κB signalling *in vitro* 40,41, but the molecular mechanism for its involvement to NF-κB signalling was not studied. A20 is an ubiquitin-editing protein that was originally identified as a TNF-inducible protein in endothelial cells and has been characterized as a central inhibitor of NF-κB signalling triggered by TNF-TNF receptor, IL-1-IL-1R, lipopolysaccharide-toll like receptor 4 and muramyl dipeptide nucleotide-binding oligomerization domain containing 2 42. Our previous work demonstrated that A20 was also required to inhibit CVB3-induced NF-κB signalling by restricting TRAF6K63-linked ubiquitination 33. Thus, we investigated whether A20 was involved in the inhibitory effect of AST-IV on NF-κB signalling. The results showed that AST-IV treatment could up-regulate and sustain the elevation of cardiac A20 expression in CVB3 mice in dose dependent manner. However, here we are still not clear which cell types express A20 in the myocardium during AST-IV treatment and whether AST-IV regulates A20 expression has tissue specificity, which will await successive studies. By overexpressed A20 through adenovirus Ad-A20 and knock down A20 through lentivirus LV-shA20 in CVB3 mice received AST-IV treatment, we found that A20 overexpression enhanced the inhibitory effect of AST-IV on NF-κB signalling, on the contrary, A20 knock down abrogate this effect, which indicated that the inhibitory effect of AST-IV on NF-κB signalling in CVB3 mice was *via* increasing A20 expression. Of course, there may other molecules involve in the inhibitory effect of AST-IV on NF-κB signalling. Undoubtedly, further studies are needed to identify the molecules which may directly interact with AST-IV in NF-κB signalling, which will be also our next work.

We further revealed that A20 was critical for anti-inflammation and therapeutic effect of AST-IV in myocarditis, evidenced by fewer inflammatory cells infiltration, better survival conditions and improved cardiac function in A20 overexpressed mice with low dose AST-IV (20 mg/kg) treatment following CVB3 infection, in contrast, more sever cardiac inflammation, lower survival rates and worsen cardiac function in A20 knock down mice even with high dose AST-IV (40 mg/kg) treatment. Our results suggested that A20 may serve as a target for screening new drugs for the treatment of VMC and may provide an insight into better analysing the effective constituents of Astragalus or other Chinese transitional herbs for exploiting optimal therapeutic strategies which may require a combination of multiple agents. Nevertheless, as for the direct use of A20 expression with adenovirus to humans, undoubtedly there still have a long way to go because of its safety and feasibility are needed to be identified.

A20 is an immediate early responsive gene and acts as negative feedback of NF-κB signalling. The transcription of A20 is itself under the control of NF-κB 36. Our data *in vitro* experiments showed that A20 mRNA was induced after CVB3 infection, but subsequently decreased quickly, implying that A20 was transiently induced following CVB3 infection, thus could not play its role for anti-inflammation, consistent with previous reports which showed that the mRNA of A20 was unstable and degraded quickly for the 3′-untranslated region (UTR) contained four copies of the canonical sequence ATTTA 43,44. Interestingly, the infected cells with AST-IV treatment sustained the elevation of the steady-state A20 expression, but AST-IV per se could not induce A20 mRNA expression. We further revealed that AST-IV had no effect on A20 transcription, since the A20 promoter activity was unchanged with AST-IV treatment. But A20 mRNA was much more stable in the presence of AST-IV, suggesting that AST-IV promoted A20 expression at posttranscriptional level by sustaining the stability of mRNA. The stability of mRNAs can be affected by miRNAs that direct the RNA-induced silencing complex (RISC) to the 3′-UTR of its targets and RNA binding proteins that control posttranscriptional gene expression 45. A recent study revealed that miR-29 prevented the RNA binding proteins HuR (human antigen R) from binding to the A20 3′-UTR and recruiting the RISC, thus protecting A20 transcripts 46. Besides, A20 transcripts were also found to be regulated by H3K4 methylation of histone at the *Tnfaip3* promoter *via* epigenetic modulation 47,48. These studies provide valuable clues for us to better understand the regulation of A20 expression.

In conclusion, our study demonstrated that AST-IV was effective for the treatment of VMC. Moreover, we uncovered a previously unknown mechanism for AST-IV because of modulating inflammatory response *via* increasing A20 expression at posttranscriptional level by stabilizing mRNA. Our findings may provide a target for screening new drugs and be helpful for exploiting optimal therapeutic strategies for VMC or other heart inflammatory diseases.
